# The Prevalence of Perineal Tears Among Women Having Spontaneous Vaginal Births with Intrapartum Fever

**DOI:** 10.3390/microorganisms13122815

**Published:** 2025-12-10

**Authors:** Manal Massalha, Eyal Rom, Ayelet Gertner Bonfis, Haya Khalilieh Suleiman, Marwa Diab, Enav Yefet, Zohar Nachum

**Affiliations:** 1 Department of Obstetrics and Gynecology, Emek Medical Center, Afula 1834111, Israel; noola87@gmail.com (M.M.); eyal_rom@hotmail.com (E.R.); yely88@gmail.com (A.G.B.); haia_kha@hotmail.com (H.K.S.); madiab6@gmail.com (M.D.); 2 The Ruth and Bruce Rappaport Faculty of Medicine, Technion-Israel Institute of Technology, Haifa 3200003, Israel; 3 Department of Obstetrics and Gynecology, Tzafon Medical Center, Poriya 1528001, Israel; 4 Azrieli Faculty of Medicine, Bar-Ilan University, Safed 1311502, Israel

**Keywords:** intrapartum fever, perineal tears, obstetric anal sphincter injury, chorioamnionitis, spontaneous vaginal delivery, maternal morbidity

## Abstract

Perineal tears are common during vaginal delivery and are associated with significant maternal morbidity. While chorioamnionitis and intrapartum fever are known to affect labor dynamics and perineal tissue integrity, their relationship with perineal trauma in spontaneous vaginal deliveries has not been established. This study aimed to evaluate the prevalence of perineal tears among women with intrapartum fever who delivered spontaneously. This retrospective cohort study included women who underwent spontaneous vaginal delivery during 2013–2021 in Israel. The study group comprised women diagnosed with intrapartum fever (≥38 °C), while afebrile women served as controls in a 1:2 ratio matched by age (<35 or ≥35 years) and gestational age (preterm/term). Perineal tears were classified according to the Royal College of Obstetricians and Gynaecologists (RCOG) criteria. Multivariable logistic regression was performed to adjust for statistically significant variables including obesity, induction of labor, epidural analgesia, amniotomy, delivery week, gestational diabetes, birth number, duration of the second stage of labor, and episiotomy. The cohort included 373 women with intrapartum fever and 746 controls. The overall rate of perineal tears was similar between febrile and afebrile women (42% vs. 40%; adjusted odds ratio [aOR] 0.99, 95% confidence interval [CI] 0.72–1.36). However, the rate of obstetric anal sphincter injury (OASIS) was lower among women with intrapartum fever (0.5% vs. 2.0%; aOR 0.10, 95% CI 0.02–0.52). Intrapartum fever was associated with higher rates of postpartum hemorrhage, manual exploration of the uterus, endometritis, anemia, and blood transfusion. Bacterial cultures were positive in 31% of febrile women, predominantly *Escherichia coli* and Group B *Streptococcus*, without association with perineal trauma. Alltogether, Intrapartum fever did not increase the risk of perineal tears in spontaneous vaginal deliveries and was paradoxically associated with a lower rate of OASIS. Further studies are warranted to explore the underlying physiological mechanisms linking temperature and perineal tissue resilience.

## 1. Introduction

Perineal tears are prevalent during birth. It is estimated that 50–79% of women may experience some form of laceration during vaginal delivery [1,2], with the majority having first- and second-degree lacerations [1,3] as classified by the Royal College of Obstetricians and Gynaecologists (RCOG) [4]. This classification grades perineal tears from 1 to 4 based on the extent of trauma to the vaginal mucosa, perineal muscles, and anal sphincter [4].

First-degree lacerations typically result in mild discomfort, whereas second-degree lacerations, which extend into the perineal muscles, are more painful and may necessitate suturing [5,6]. Both may lead to considerable complications, such as pain and interference with daily activities and sexual dysfunction [5]. Pain generally diminishes over time, yet, it can significantly impact activities of daily living, such as sitting, mobility, and personal hygiene, during the early postpartum period. Even minor tears may temporarily hinder maternal caregiving and routine functions [7]. Dyspareunia is uncommon with first-degree lacerations but may be present with second-degree tears [7]. Spontaneous perineal lacerations were associated with elevated risk for pelvic organ prolapse to or beyond the hymen in up to 10 years follow-up [8].

Severe perineal tears (grades 3–4) are classified as obstetric anal sphincter injuries (OASIS) and require advanced repair by a specialist [2]. OASIS may cause significant physical and social implications. Long-term effects include anal incontinence, discomfort, and sexual dysfunction. The significant clinical sequel and its associated ramifications have imposed a substantial burden on healthcare professionals and systems [9].

Several risk factors for OASIS have been identified [4], including Asian ethnicity, nulliparity, macrosomia, shoulder dystocia, occipito-posterior position, protracted second stage of labor, and instrumental delivery.

Chorioamnionitis is a common condition affecting 2–5% of all births [10]. Any maternal temperature between 38.0 °C and 38.9 °C without any additional risk factors is considered isolated maternal fever, whereas clinical chorioamnionitis is diagnosed when the maternal temperature is greater than or equal to 39.0 °C or between 38.0 °C and 38.9 °C with one additional clinical risk factor present such as maternal tachycardia, uterine tenderness, maternal leukocytosis, malodorous vaginal discharge or amniotic fluid, and fetal tachycardia [10,11,12,13,14,15,16,17]. A retrospective study evaluated risk factors for OASIS during instrumental delivery and discovered that chorioamnionitis was a significant risk factor (adjusted odds ratio (aOR) 2.2; 95% confidence interval (CI) 1.1–4.4) [18].

Chorioamnionitis or intrapartum fever may increase the risk of OASIS, likely due to their association with prolonged labor [6].Furthermore, it is probable that intrapartum fever adds to tissue inflammation, resulting in diminished skin integrity and hence more significant trauma. In addition, it has been previously shown that administering antibiotics for intrapartum fever decreases the risk of infection and wound breakdown associated with OASIS in third- and fourth-degree lacerations [19,20]. Thus, a presence of inflammation in the pelvic floor may lead to increased risk of perineal tears.

While many risk factors for perineal tear are well established, and specifically chorioamnionitis for higher-order tears during operative delivery, such association regarding spontaneous vaginal deliveries has not been evaluated yet.

This study aimed to evaluate the prevalence of perineal tears among women with intrapartum fever who delivered spontaneously.

## 2. Materials and Methods

This retrospective cohort study was conducted at Emek Medical Center in Afula, Israel, a university teaching hospital, with a delivery unit that accommodates about 5000 deliveries per year. The institutional review board approved the trial protocol (115-22-EMC). Informed consent was not necessary for this study.

The study cohort included women who had spontaneous vaginal deliveries between August 2013 to September 2021. The research group consisted of women who had spontaneous vaginal delivery and diagnosed with intrapartum fever. The threshold for intrapartum fever was defined as maternal temperature ≥38 °C (≥100.4 °F) orally [11,21]. In these cases, a fever workup was performed, including bacterial cultures from blood, urine, and products of conception (placenta and membranes). Intravenous antibiotics were administered according to the clinical scenario: isolated maternal fever was treated with *group B streptococcus* prophylaxis using penicillin G or clindamycin, while suspected chorioamnionitis was managed with a broad-spectrum antibiotic regimen consisting of gentamicin, ampicillin, and metronidazole. After delivery, additional one dose of antibiotics was administered. If fever persisted postpartum, antibiotics were administered for at least 7 days. In the control group we included those who had spontaneous vaginal delivery without intrapartum fever. The control group was chosen randomly during the same study period. Research and control groups were matched by maternal age < 35 and at least 35 years and preterm and term delivery in a 1:2 ratio.

Perineal tears and OASIS were diagnosed and graded according to the RCOG classification [4], by a physician in the delivery ward.

Patients who underwent cesarean delivery, had multifetal pregnancies, or underwent operative vaginal delivery were excluded from the cohorts.

### 2.1. Study Outcomes

The primary outcome was the rate of perineal tears. Secondary outcomes included obstetric complications, the rates of each perineal tear grade, the bacterial species identified in positive cultures (blood, urine, and products of conception), and the composition of bacterial growth. Study groups’ characteristics, obstetric data, clinical outcomes and laboratory data were collected using the Chameleon electronic medical records (EMR) system, a comprehensive digital platform routinely used for documentation of obstetric admissions, procedures, and outcomes.

### 2.2. Statistical Analysis

Assuming that intrapartum fever would increase the risk for perineal tear from 50% in the controls without intrapartum fever [2], to 60% in the research group, 999 women were needed for both groups in 2:1 ratio (5% two sided alpha, 85% power).

Groups were compared using the student t-test (or the Wilcoxon two-sample test) for continuous variables and χ^2^ (or Fisher’s exact test) for categorical variables. Controlling for different groups’ background characteristics was done with multivariable logistic regression for statistically significant outcomes in univariable analysis. All the analyses were performed using SAS 9.4 software (SAS Institute Inc., Cary, NC, USA).

## 3. Results

We included 373 women with spontaneous vaginal deliveries in the research group and 746 in the control group. Obstetrics and demographic variables of both groups are presented in Table 1. The research group had higher body mass index (BMI), and an earlier gestational age at delivery. Additionally, the research group showed a higher rate of labor induction and amniotomy, longer time from rupture of membranes to delivery, longer second stage of labor and a greater use of epidural analgesia. Slightly higher neonatal birth weight was documented in the research group.

Univariable and multivariable analyses of maternal outcomes are presented in Table 2. The incidence of perineal tears was similar between the research and the control groups in univariable analysis [158 (42%) vs. 298 (40%), respectively; *p* = 0.44] and also after multivariable logistic regression adjusting for obesity, labor induction, epidural analgesia, amniotomy, delivery week, gestational diabetes mellitus, birth number, time from rupture of membranes to delivery, second stage of labor duration, episiotomy and birth weight (aOR 0.99, 95% CI 0.72–1.36). There was no difference in the rate of grade 1 and grade 2 tears between the groups. Conversely, the rate of OASIS (third or fourth degree perineal tears) was lower with marginal significance in the research group compared to controls in univariable analysis (2 (0.5%) versus 15 (2.0%), respectively, *p* = 0.057) and became statistically significant after controlling for the confounders mentioned above (aOR 0.1 95% CI 0.02–0.52).

The following obstetrical complications were higher in the research group compared to the control group in univariable analysis and also after adjusting for the confounders mentioned above; postpartum hemorrhage, manual exploration of the uterine cavity, endometritis, anemia, the rate of intravenous iron sucrose treatment and the need for blood transfusion (Table 2).

Table 3 presents the results of bacterial cultures obtained from products of conception, blood, and urine samples. A total of 337 (90%) women in the research group were cultured. Of them, 106 (31%) women had at least one positive culture. Among the cases with positive cultures, 45 (43%) had associated perineal tears, whereas tears were present in 101 cases (44%) with negative cultures (*p* = 0.83).

Among 154 positive cultures, 109 (71%) were isolated from products of conception, 23 (15%) from blood, and 22 (14%) from urine. Among the isolates from the products of conception, *Escherichia coli* and *Group B Streptococcus* were the most frequently identified organisms (Table 3).

## 4. Discussion

In the present study we aimed to evaluate the prevalence of perineal tears among women with intrapartum fever who delivered spontaneously. The study’s results indicate that the prevalence of perineal tears is comparable between women who had intrapartum fever and those remaining afebrile during labor. Conversely, the rate of OASIS was lower in the research group after controlling for background characteristics. Intrapartum fever was associated with increased maternal morbidity in the peripartum period, mainly postpartum hemorrhage, manual exploration of uterine cavity, endometritis, anemia, and the need for blood transfusion.

Fever during labor can adversely affect the course and outcome of childbirth by disrupting normal uterine physiology and increasing the likelihood of uterine contractile dysfunction. This impaired contractility, in turn, elevates the risk of both cesarean delivery and operative vaginal delivery, as consistently demonstrated in prior studies [22,23,24]. In addition to influencing delivery mode, intrapartum fever has been associated with a constellation of labor abnormalities, including prolonged labor duration and increased rates of obstetric interventions performed for failure to progress. These challenges often reflect the interplay between maternal systemic inflammatory responses and the myometrium’s diminished ability to generate effective contractions. Furthermore, intrapartum fever contributes to heightened maternal morbidity. Women who develop fever are more likely to experience postpartum hemorrhage, which may arise from uterine atony secondary to labor dystocia or prolonged exposure to inflammatory mediators. Notably, the risk of these complications appears to intensify as both the severity and the duration of the fever increase. Higher peak temperatures and longer periods of elevated maternal temperature are independently associated with worse obstetric outcomes, suggesting a dose–response effect. This underscores the importance of careful monitoring, timely identification of intrapartum fever, and appropriate management to mitigate its potential impact on labor progression and maternal well-being [22,23,24].

The precise prevalence of OASIS is not definitively established, but epidemiological data suggest an incidence ranging from 0.1% to 2.1% [25,26,27]. A Similar occurrence was observed in our study. Chorioamnionitis was found to be an independent risk factor for OASIS during instrumental delivery [18]. However, in our study which focused on spontaneous vaginal deliveries, fever was found to have a protective effect on the risk for OASIS.

Our study demonstrated a significantly longer second stage of labor in the research group, suggesting that intrapartum fever may contribute to labor dystocia or altered labor physiology. Evidence from previous investigations supports the clinical relevance of these findings. In a study specifically evaluating the consequences of prolonged second-stage labor, researchers found a clear, stepwise relationship between second-stage duration and adverse obstetric outcomes. As the second stage lengthened, the rates of operative vaginal delivery and severe perineal trauma increased accordingly, highlighting the cumulative risk associated with protracted labor. When examining duration-specific subgroups, the study reported that the incidence of clinical chorioamnionitis rose markedly once the second stage exceeded 12 h, compared with the reference group experiencing 2 to 6 h of second-stage labor (adjusted odds ratio [aOR] 4.9, 95% confidence interval [CI] 1.6–14.8). Similarly, the probability of sustaining severe perineal tears increased substantially in the 6- to 12-h subgroup relative to the 2- to 6-h group (aOR 8.1, 95% CI 1.6–42.6). These findings underscore the strong association between prolonged labor, maternal infectious morbidity, and pelvic floor injury. Importantly, although prolonged labor is known to be closely linked with intrapartum fever, the referenced study did not directly evaluate the association between maternal fever and the occurrence of severe perineal tears [28]. To our knowledge, the rate of perineal trauma specifically among parturients with fever during labor has not been quantified directly in the existing medical literature. This gap highlights an opportunity for future research to more precisely characterize the interplay between intrapartum fever, labor progression, and perineal injury.

To our knowledge this is the first study demonstrating that fever during labor was associated with less OASIS. The reason for that is not clear. The available evidence consistently showed that intrapartum fever was associated with adverse maternal outcomes, including increased rates of cesarean and operative deliveries, postpartum hemorrhage, and infection, but there was no reported association with a reduction in OASIS [24].

The relationship between perineal temperature and the likelihood of perineal trauma has been explored predominantly through studies evaluating the application of warm compresses during the second stage of labor. Rather than examining fever or elevated maternal temperature as a risk factor, the available evidence focuses on therapeutic warming of the perineal tissues as a strategy to reduce OASIS and other forms of perineal damage. Warm compresses are thought to enhance tissue elasticity, promote relaxation of the pelvic floor, and reduce the likelihood of uncontrolled perineal stretching during crowning. Multiple randomized trials and meta-analyses have consistently shown that applying warm, moist compresses to the perineum during and between pushing efforts is associated with improved perineal outcomes. A systematic review and meta-analysis by Magoga et al. [29], which synthesized data from seven randomized controlled trials including 2103 participants, demonstrated a meaningful reduction in severe perineal trauma among women receiving warm compresses. In this analysis, the intervention significantly increased the likelihood of achieving an intact perineum (22.4% vs. 15.4%; RR 1.46, 95% CI 1.22–1.74) and markedly reduced the incidence of both third-degree tears (1.9% vs. 5.0%; RR 0.38, 95% CI 0.22–0.64) and fourth-degree tears (0.0% vs. 0.9%; RR 0.11, 95% CI 0.01–0.86). When third- and fourth-degree tears were combined, the protective effect of warm compresses remained substantial (RR 0.34, 95% CI 0.20–0.56). Additionally, episiotomy rates were lower in the intervention group (10.4% vs. 17.1%; RR 0.61, 95% CI 0.51–0.74), suggesting a broader effect on reducing perineal trauma and the need for operative perineal interventions. More recent evidence further strengthens these observations. Maghalian et al. [30] conducted a comprehensive review including both randomized and non-randomized trials, encompassing 14 studies in total. Their meta-analysis confirmed that warm perineal compresses significantly reduce second degree perineal tears (RR 0.65, 95% CI 0.54–0.79; *p* < 0.00001), third degree perineal tears (RR 0.32, 95% CI 0.15–0.67; *p* = 0.003), and fourth degree perineal tears (RR 0.11, 95% CI 0.01–0.87; *p* = 0.04) and decrease postpartum perineal pain (RR 0.23, 95% CI 0.08–0.66; *p* = 0.0006). The intervention also lowered episiotomy rates (RR 0.63, 95% CI 0.46–0.86) while substantially increasing the probability of an intact perineum (RR 3.06, 95% CI 1.79–5.22). Importantly, this review demonstrated that the beneficial effect of warm compresses was consistent across different populations, including variations in parity and ethnicity, and was supported by trial sequential analysis and GRADE assessment.

These findings are consistent with earlier Cochrane reviews [31], which concluded that warm compresses offer moderate-quality evidence for reducing third- and fourth-degree tears. Collectively, the literature suggests that controlled perineal warming during the second stage of labor is a simple, low-cost intervention that meaningfully reduces severe perineal trauma and improves maternal postpartum recovery.

It was hypothesized that the mechanism by which hot compresses prevent perineal tears during the second stage of labor involves several physiological effects. Warm compresses applied to the perineum increase local blood flow and tissue elasticity, which helps the perineal tissues stretch more effectively during childbirth. This increased elasticity reduces the likelihood of tears, particularly severe ones such as third- and fourth-degree lacerations. The application of warm compresses also helps to relax the perineal muscles, which can reduce the resistance against the descending fetal head. This relaxation minimizes the stress and strain on the perineal tissues, further decreasing the risk of tearing. Additionally, the warmth from the compresses can provide a soothing effect, potentially reducing maternal discomfort and allowing for a more controlled and less forceful delivery [30,31]. Additional studied are required to further evaluate those suggested mechanisms.

Gestational diabetes mellitus was significantly more associated with intrapartum fever, a finding that aligns with growing interest in understanding the broader infectious and inflammatory implications of gestational diabetes mellitus during labor and delivery. In our earlier work, we examined this relationship more comprehensively by evaluating the association between gestational diabetes mellitus and the incidence of peripartum infections—specifically chorioamnionitis and/or endometritis—in a large retrospective cohort study [32]. That investigation included a robust sample of 1683 women diagnosed with gestational diabetes mellitus and an equal number of 1683 matched controls without gestational diabetes mellitus. Matching was carefully performed based on clinically relevant maternal characteristics, including advanced maternal age ≥35 years, primiparity, pre-gestational BMI, mode of delivery (cesarean and vacuum-assisted), and preterm birth, to ensure comparability between groups and minimize confounding. The primary outcome of interest was the overall rate of peripartum infections. Despite biologically plausible mechanisms linking gestational diabetes mellitus with altered immune function and increased susceptibility to infection, our analysis revealed no significant differences between the gestational diabetes mellitus and non—gestational diabetes mellitus groups in the aggregate rate of peripartum infectious morbidity (26 [1.5%] vs. 14 [0.8%], *p* = 0.056). Similarly, the incidence of specific infectious diagnoses—chorioamnionitis, endometritis, or other defined infection-related outcomes—did not significantly differ. These findings persisted even after rigorous adjustment for additional potential confounders, including epidural analgesia, maternal BMI, maternal age, hypertensive disorders of pregnancy, and gestational age at delivery, using multivariable logistic regression. The adjusted analysis yielded an odds ratio of 1.7 (95% CI 0.9–3.4), further supporting the absence of a meaningful association between gestational diabetes mellitus and peripartum infection risk.

Given these consistent results, we propose that the increased frequency of intrapartum fever observed in the current study may not reflect true infectious morbidity associated with gestational diabetes mellitus but instead represent confounding through labor-related factors. Women with gestational diabetes mellitus are more likely to experience higher birthweights, require induction of labor, and undergo longer labor courses—all of which may contribute independently to the development of intrapartum fever through prolonged membrane rupture, increased examinations, or heightened inflammatory response. Importantly, the rate of preeclampsia, another condition associated with higher induction rates and potential infectious risk, did not significantly differ between the study groups. This lack of difference is likely attributable to the low overall prevalence of preeclampsia (2%) in both cohorts, limiting its influence on the observed outcomes. Collectively, these observations reinforce our prior conclusion that gestational diabetes mellitus alone does not appear to elevate the risk of peripartum infections, and they highlight the complex interplay between metabolic conditions, obstetric management, and labor-associated inflammatory processes.

This study possesses several methodological strengths. Importantly, it is the first to investigate the association between intrapartum fever and perineal lacerations specifically in spontaneous vaginal deliveries, thereby addressing a gap in the current literature, which has largely concentrated on operative deliveries. The inclusion of microbiological cultures from pertinent clinical specimens provided additional data, although no significant association between infectious etiology and perineal trauma was observed.

However, the study is limited by its retrospective design and there is a risk of bias due to potential confounders that were not evaluated. The protective effect of fever on OASIS was a secondary outcome in this study, which was not powered to evaluate this outcome. A sample size of 1720 women would be required to demonstrate a statistically significant difference when the incidence of OASIS is 0.5% versus 2%, whereas our cohort comprises only 65% of the required sample size.

In conclusion, while intrapartum fever remains a significant concern for other maternal and neonatal outcomes, our data do not support its role as an independent risk factor for perineal lacerations and a protective affect might be present for OASIS. Further studies are warranted to explore the underlying physiological mechanisms linking temperature and perineal tissue resilience.

## Figures and Tables

**Table 1:** Demographic and obstetric variables of women according to study group.

Variables	Research Group N = 373	Control Group N = 746	*p* Value
Age (years)	26.4 ± 4.5	26.5 ± 5.2	0.68
BMI before pregnancy (kg/m^2^)	24.0 ± 4.5	23.5 ± 4.8	0.08
Obesity BMI > 30	28 (8%)	21 (3%)	0.0003
Ethnicity			
Jews	203 (54%)	370 (50%)	0.13
Arabs	170 (46%)	376 (50%)	
Smoking	11 (3%)	32 (4%)	0.30
Pregnancy number	1.7 ± 1.2	1.8 ± 1.2	0.03
Birth number	1.3 ± 0.7	1.5 ± 1.0	0.03
Delivery week	38.9 ± 2.0	39.3 ± 1.8	0.0002
Hypothyroidism	19 (5%)	31 (4%)	0.47
Pregestational diabetes mellitus	3 (0.8%)	6 (0.8%)	1
Gestational diabetes mellitus	42 (11%)	50 (7%)	0.008
Chronic hypertension	4 (1%)	15 (2%)	0.25
Gestational hypertension	11 (3%)	33 (4%)	0.23
Preeclampsia	7 (2%)	18 (2%)	0.57
Proteinuria	8 (2%)	8 (1%)	0.20
Polyhydramnios	6 (2%)	7 (1%)	0.38
Oligohydramnios	17 (5%)	24 (3%)	0.26
Placental abruption	4 (1%)	6 (1%)	0.74
Induction of labor	234 (63%)	264 (35%)	<0.0001
Epidural	293 (79%)	292 (39%)	<0.0001
Amniotomy	206 (55%)	242 (32%)	<0.0001
Time from rupture of membrane to delivery (hours)	14.2 ± 20.4	7.7 ± 12.4	<0.0001
Second stage of labor duration (min)	100 ± 81	46 ± 61	<0.0001
POP presentation	1 (0.3%)	2 (0.3%)	1
Fetal birth weight	3292 ± 497	3198 ± 475	0.002
Fetal gender			
Male	206 (55%)	380 (51%)	0.18
Female	167 (45%)	366 (49%)	
Meconium	75 (20%)	86 (12%)	0.0001

Values are given as the mean (standard deviation) or number (percent). BMI, body mass index; POP, persistent occipito-posterior. Groups were compared using the student *t*-test (or the Wilcoxon two-sample test) for continuous variables and χ^2^ (or Fisher’s exact test) for categorical variables.

**Table 2:** Primary and secondary outcomes.

Outcomes	Research Group N = 373	Control Group N = 746	*p* Value	aOR 95% CI
Any tear	158 (42%)	298 (40%)	0.44	0.99 [0.72–1.36]
First degree perineal tear	74 (20%)	146 (20%)	0.92	
Second degree perineal tear	84 (23%)	154 (21%)	0.47	
Third/fourth degree perineal tear (OASIS)	2 (0.5%)	15 (2.0%)	0.057	0.1 [0.02–0.52]
Episiotomy	80 (21%)	109 (15%)	0.004	
Postpartum hemorrhage	49 (13%)	43 (6%)	<0.0001	2.1 [1.3–3.6]
Manual exploration of uterine cavity	37 (10%)	38 (5%)	0.002	1.9 [1.1–3.4]
Hemoglobin before labor (gr%)	12.1 ± 1.2	12.1 ± 1.2	0.91	
Hemoglobin at discharge (gr%)	10.9 ± 1.7	11.6 ± 1.6	<0.0001	
Delta hemoglobin (gr%)	1.2 ± 1.5	0.5 ± 1.1	<0.0001	
Anemia (hemoglobin < 11.0 gr%) **	96 (26%)	32 (4%)	<0.0001	6.7 [4.1–10.9]
IV Iron sucrose treatment *	64 (17%)	73 (10%)	0.0004	1.7 [1.1–2.6]
Blood transfusion	23 (6%)	14 (2%)	0.0002	3.4 [1.5–7.8]
Postpartum infections ***				
Endometritis	8 (2.1%)	1 (0.1%)	0.0009	55 [5.6–507]
Postpartum urinary tract infection	12 (3%)	15 (2%)	0.22	
Postpartum fever of unknown cause	0 (0)	3 (0.4%)	0.22	
Postpartum urinary retention	12 (3%)	16 (2%)	0.28	

Data are the mean ± standard deviation or number (percent). * Administered in hemoglobin ≤ 9.5 g%. ** According to hemoglobin at discharge (gr%). *** Signs and symptoms indicative of infection were documented after delivery. aOR, adjusted odds ratio—was adjusted for obesity, labor induction, epidural analgesia, amniotomy, delivery week, gestational diabetes mellitus, birth number, time from rupture of membranes to delivery, second stage of labor duration and episiotomy. Multivariable analysis was done for statistically significant outcomes in univariable analysis. OASIS, obstetric anal sphincter injuries.

**Table 3:** Bacterial culture profile.

Bacteria Species	Products of Conception Culture N = 109	Blood Culture N = 23	Urine Culture N = 22
*Aerococcus viridans*	1	0	0
*Bacillus cereus*	1	0	0
*Bacillus *sp.	0	2	0
*Bacteroides fragilis*	1	0	0
*Bacteroides vulgatus*	1	0	0
*Candida glabrata*	1	0	0
*Candida glabrata (T. glab.)*	1	0	0
*Citrobacter koseri*	2	0	0
*Enterobacter cloacae*	1	0	0
*Enterococcus faecalis*	6	1	2
*Enterococcus *sp. *(group D)*	1	0	0
*Escherichia coli*	24	0	10
*Group B Streptococcus*	23	3	4
*Haemophilus influenzae*	1	1	0
*Klebsiella pneumoniae*	5	0	3
*Lactobacillus jensenii*	2	0	0
*Lactobacillus *sp.	5	0	2
*Listeria monocytogenes*	1	0	0
*Micrococcus luteus*	0	1	0
*Morganella morganii*	2	1	0
*Mycoplasma hominis*	1	0	0
*Proteus mirabilis*	2	0	0
*Pseudomonas aeruginosa*	1	0	1
*Staphylococcus aureus*	3	2	0
*Staphylococcus coag. neg. group*	3	3	0
*Staphylococcus epidermidis*	1	1	0
*Staphylococcus haemolyticus*	1	1	0
*Staphylococcus hominis*	2	2	0
*Staphylococcus lugdunensis*	1	0	0
*Streptococcus anginosus*	2	0	0
*Streptococcus mitis (viridans)*	8	1	0
*Streptococcus oralis*	0	3	0
*Streptococcus pneumoniae*	0	1	0
*Ureaplasma urealytic*	5	0	0

## Data Availability

The original contributions presented in this study are included in the article. Further inquiries can be directed to the corresponding authors.
